# The Canadian Urban Environmental Health Research Consortium – a protocol for building a national environmental exposure data platform for integrated analyses of urban form and health

**DOI:** 10.1186/s12889-017-5001-5

**Published:** 2018-01-08

**Authors:** Jeffrey R. Brook, Eleanor M. Setton, Evan Seed, Mahdi Shooshtari, Dany Doiron, CANUE – The Canadian Urban Environmental Health Research Consortium, Philip Awadalla, Michael Brauer, Howard Hu, Kim McGrail, Dave Stieb, Padmaja Subarrao, Paul Demers, Doug Manuel, John McLaughlin, Chris Carlsten, Meghan Azad, Stephanie Atkinson, Rick Burnett, Wendy Lou, Daniel Rainham, Greg Evans, Ray Copes, Olimpia Pantelimon, Audrey Smargiassi, Hugh Davies, Paul Villeneuve, Matilda van den Bosch, Diane Chaumont, Johannes Feddema, Tim Takaro, Amir Hakami, Markey Johnson, Marianne Hatzopoulou, Ahsan Habib, Daniel Fuller, Michael Widener

**Affiliations:** 1 Processes Research Section, Air Quality Research Division, Environment and Climate Change Canada, Toronto, ON Canada; 2 Dalla Lana School of Public Health, University of Toronto, Toronto, Canada; 3 Geography Department, University of Victoria, Victoria, Canada; 4 Research Institute of McGill University Health Centre, Montreal, Canada

**Keywords:** Urban form, Exposure, Air pollution, Noise pollution, Green space, Transportation, Climate, Public health

## Abstract

**Background:**

Multiple external environmental exposures related to residential location and urban form including, air pollutants, noise, greenness, and walkability have been linked to health impacts or benefits. The Canadian Urban Environmental Health Research Consortium (CANUE) was established to facilitate the linkage of extensive geospatial exposure data to existing Canadian cohorts and administrative health data holdings. We hypothesize that this linkage will enable investigators to test a variety of their own hypotheses related to the interdependent associations of built environment features with diverse health outcomes encompassed by the cohorts and administrative data.

**Methods:**

We developed a protocol for compiling measures of built environment features that quantify exposure; vary spatially on the urban and suburban scale; and can be modified through changes in policy or individual behaviour to benefit health. These measures fall into six domains: air quality, noise, greenness, weather/climate, and transportation and neighbourhood factors; and will be indexed to six-digit postal codes to facilitate merging with health databases. Initial efforts focus on existing data and include estimates of air pollutants, greenness, temperature extremes, and neighbourhood walkability and socioeconomic characteristics. Key gaps will be addressed for noise exposure, with a new national model being developed, and for transportation-related exposures, with detailed estimates of truck volumes and diesel emissions now underway in selected cities. Improvements to existing exposure estimates are planned, primarily by increasing temporal and/or spatial resolution given new satellite-based sensors and more detailed national air quality modelling. Novel metrics are also planned for walkability and food environments, green space access and function and life-long climate-related exposures based on local climate zones. Critical challenges exist, for example, the quantity and quality of input data to many of the models and metrics has changed over time, making it difficult to develop and validate historical exposures.

**Discussion:**

CANUE represents a unique effort to coordinate and leverage substantial research investments and will enable a more focused effort on filling gaps in exposure information, improving the range of exposures quantified, their precision and mechanistic relevance to health. Epidemiological studies may be better able to explore the common theme of urban form and health in an integrated manner, ultimately contributing new knowledge informing policies that enhance healthy urban living.

## Background

Multiple external environmental exposures related to residential location and urban form including, air pollutants [1–3], noise [4–6], greenness [7], and walkability [8–10] have been linked to health impacts or benefits. In Canada, more than 80% of the population lives in urban areas [11], and with clear evidence that health impacts can occur even at exposure levels that are considered to be low [12], there is an urgent need to learn how to design and modify cities to improve, not degrade, population health [13]. A concerted effort to address this need could provide the informative science to support urban planners and population health-related policy makers who are faced with very real issues such as, urban sprawl, traffic congestion, car-dependency, social equity and sustainability.

We hypothesize that a coordinated program capitalizing on: 1)the opportunity of emerging big data relating to our physical environment; 2) improvements in methods for managing and analyzing large data streams; 3) learning from efforts to increase power for epidemiological discovery by initiating large prospective cohorts [14–17], combining existing cohorts [18] or building large administrative cohorts [19–21]; can support the production of substantial new knowledge about how the environment contributes to chronic disease. Hu et al. (2017) suggested that population health stands to benefit from the big data and precision medicine agendas if a parallel effort to introduce measures that capture potential health risks at multiple levels of influence can be realized [22]. We view such an effort as bringing ‘big environmental data’ into the equation and the insights gained could have applications from the individual to the population level [23].

In 2015 the Canadian Institutes of Health Research (CIHR) called for a new national consortium that would bring together scientific and other expertise from a wide variety of disciplines and fields from academia, government, non-governmental organizations and industry to focus on specific research priorities that can only be addressed through interdisciplinary and intersectoral research. This included developing a ‘data and methodological hub’ where environmental researchers could collaborate with cohorts and health researchers on focused health projects using innovative measurement models and ‘analysis-ready’ data [24]. Responding to this call, the Canadian Urban Environmental Health Research Consortium (CANUE) was established and aims, through a coordinated program, to capitalize on Canada’s growing big data capacity by facilitating the linkage of extensive geospatial exposure data to the wealth of established cohorts and administrative health data holdings (http://canue.ca). This linkage will enable investigators to test a variety of hypotheses related to the interdependent associations of built environment features with diverse health outcomes encompassed by the cohorts and administrative data.

The goal of this paper is to present CANUE’s protocol for acquiring, developing and indexing exposure data for integration with health databases, and to discuss some of the challenges associated with developing accurate exposure estimates related to urban form. In addition, we provide examples of plans and opportunities to generate big environmental data to advance our understanding of environmental health and help optimize urban planning to benefit public health.

## Methods

### Data protocol

**Fig. 1:**
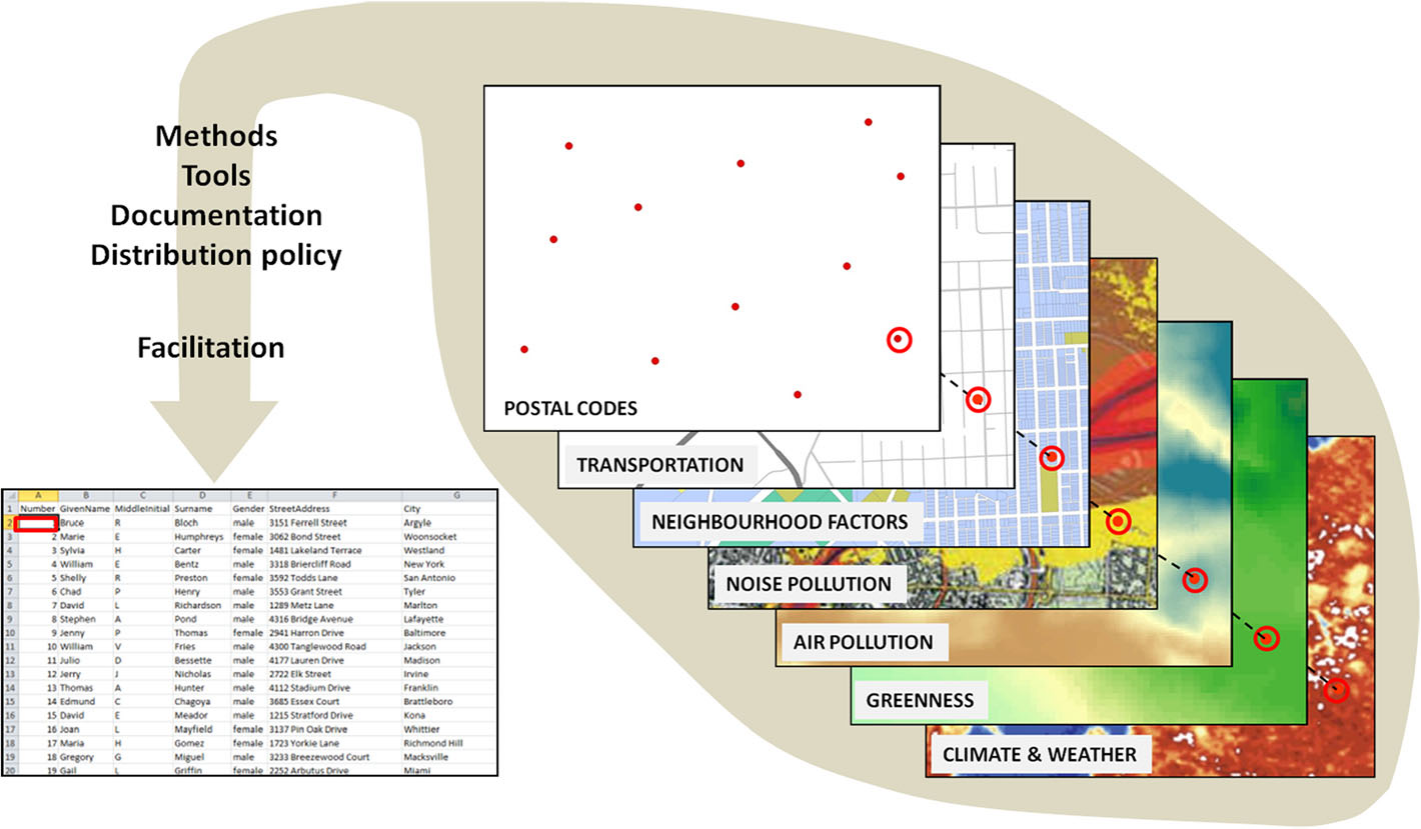
Schematic of the main data products and linkages being compiled through CANUE

CANUE’s vision is to increase scientific understanding of the interactions among the physical features of the urban environment and health. This understanding will lead to cost-effective actions that promote healthy childhood development and aging, reduce the burden of chronic disease, and minimize the impact of changing environments. To achieve this vision, CANUE is establishing and implementing a protocol for compiling environmental measures or metrics that: quantify exposure, behaviour patterns or effect modifiers; vary spatially on the urban and suburban scale; can be obtained for multiple urbanized regions in Canada and; could be modified through changes in policy or individual behaviour to benefit health. While urban areas are the focus, exposures across rural Canada are also being compiled. CANUE’s main data-related products are: (1) multiple spatial maps or surfaces (Fig. 1), one for each environmental exposure metric, that can linked to individuals included in health studies based upon the locations where they spend time (e.g., home); (2) computational tools to derive new exposure metrics; (3) documentation of the methods and data, how they were derived, by whom and key information relevant to data usage (e.g., potential limitations) and; (4) facilitation of exposure data extraction for individual-level data merging with major Canadian health databases (Table 1), including consideration of residential mobility.

**Table 1:** Major Canadian Health Databases

Type	Name	Participants	Start Year
Cohort	Canadian Partnership for Tomorrow Project	300,000	2000–2009
Cohort	Canadian Longitudinal Study on Aging	50,000	2010
Cohort	Canadian Healthy Infant Longitudinal Development Study	10,059	2008
Cohort	All Our Babies and All Our Families	6774	2008
Cohort	Alberta Pregnancy Outcomes and Nutrition	5841	2009
Cohort	TARGet Kids!	5062	2008
Cohort	Ontario Birth Study	2748	2013
Cohort	3D Study - Design, Develop, Discover	2456	2010
Cohort	Maternal-Infant Research on Environmental Chemicals	2000	2008
Cohort	Canadian Cohort of Obstructive Lung Disease	1400	2009
Cohort - administrative	Canadian Census Health and Environment Cohort (1991)	2,500,000	1991
Cohort - administrative	Canadian Census Health and Environment Cohort (1996)	3,500,000	1996
Cross-sectional survey	Canadian Health Measures Survey	23,000	2007–2015
Linked Administrative Database	Population Data BC	> 5,000,000	1980s
Linked Administrative Database	Manitoba Centre for Health Policy	> 1,000,000	1970s
Linked Administrative Database	Institute for Clinical Evaluative Science	13,000,000	1986
Network (26 pregnancy and birth cohorts)	Research Advancement Through Cohort Cataloguing and Harmonization	~ 125,000	varies

**Fig. 2:**
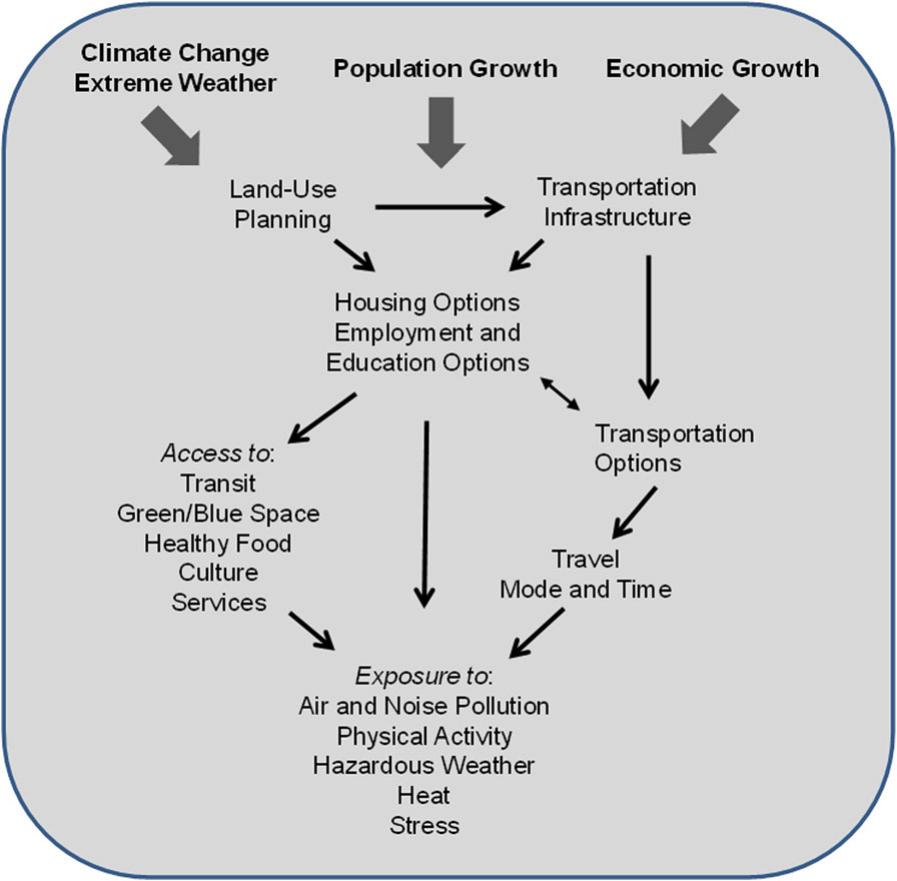
Relationships among factors associated with urban form and individual behaviours and environmental exposures. Land-use planning controls the over-arching modifiable features of the urban environment and, in addition to responding to external forces associated with population and economic growth and local weather, including extreme events and climate change, can potentially be optimized to have the greatest benefit to public health

CANUE currently focuses on collating and generating exposure metrics in six domains: Air Pollution, Noise, Greenness, Weather and Climate, Transportation, and Neighbourhood Factors, which include land-use, urban design and social determinants. These factors are grouped together, recognizing that much of our health and well-being begins at the neighbourhood level and there has been a great deal of theoretical guidance as to which factors at this scale are paramount, influencing key behaviours such physical activity and diet [25, 26]. Also, it is at this scale that patterns in socioeconomic factors manifest, creating a backdrop of individual susceptibility that must be considered in the context of public health. Active within CANUE are domain-specific working groups assessing the state of knowledge and research nationally and internationally, identifying critical gaps and conducting strategic research to improve the available exposure metrics. Fig. 2 places the six domains in the context of key external forces influencing urban form: population growth, economic growth, and weather/climate which includes factors such as extreme heat and cold events and longer-term climate change. In general, the main public responses to these forces are land-use planning and transportation infrastructure decisions; in turn, this leads to individual options around housing, employment and education locations. Choices made based upon these options or constraints subsequently impact an individual’s access to or interaction with urban features of health relevance and dictates individual behaviour such as time spent commuting and working or time available for leisure and family. All ultimately impact the magnitude of a range of harmful or beneficial exposures and thus individual and public health.

The exposure data or metrics being compiled in CANUE are georeferenced at the six-digit postal code level (or other geographic level as appropriate) facilitating linkage with health research cohorts and administrative health databases. Changes in the geographic distribution of exposure over time are important to consider given the potential time windows over which environmental factors can contribute to adverse health outcomes and chronic disease development. The temporal resolution required and the number of years back in time for which exposures can be estimated varies across the domains based upon the rate of change over time and available data sources. Accurately accounting for short and long term exposure time windows represents a considerable challenge and will be discussed below.

### Compilation of existing exposure information

Within each domain, existing data are being centralized to improve accessibility for researchers and subsequent integration with Canadian health data platforms. Work with these initial datasets (Table 2) is also facilitating development of CANUE’s infrastructure for data transfers, storage, manipulation into analysis-ready formats and documentation, including terms of use that assure requirements of the data originators are respected. This initial phase is helping to identify challenges related to harmonization of environmental data within and between domains and with similar efforts internationally. Several of these existing exposure datasets have been previously used in health research, nationally or regionally within Canada and are thus in analysis-ready format; while others require further processing and quality assessment before linkage with health data.

**Table 2:** Existing metrics

Existing Metrics	Geographic Extent	Spatial Resolution	Time Periods	Description	Ref.
Air Pollution					
Surface concentrations of PM_2.5_	National	1 km	1998 to present	Satellite-derived annual mean concentration	[27
Ambient concentrations of NO_2_	National	<100 m	1984–2012	National land use regression model of annual mean concentration, based on 2011 data and adjusted for historical estimates	[28]
Ambient concentrations of NO_2_	National	<100 m	1984–2006	National model of annual mean concentration based on 10 city-specific land use regression models (field monitoring between 2002 and 2010) and adjusted using observational data for each year	[29]
Ambient concentrations of O_3_	National	10 km - 21 km	2003 - present	Air quality forecast-based estimate, adjusted with surface observation data - annual and monthly average concentration	[30]
Ambient concentrations of SO_2_	National	30 km	2005–2015	Air quality model-based estimate of 3-year running annual average concentration	[32]
Noise Pollution					
A-weighted sound pressure level and related summary metrics	Regional (Montreal)	10 m	~ 2014	Land use regression-based model based on field monitoring conducted 2010–2014	[33]
A-weighted sound pressure level and related summary metrics	Regional (Toronto)	10 m	~ 2013	Land use regression-based model based on field monitoring conducted 2012–2013	[34]
A-weighted sound pressure level and related summary metrics	Regional (Vancouver)	10 m	2003	Sound propagation model (CadnaA)	[36]
Greenness					
Normalized Difference Vegetation Index	National +	30 m	1985 to present	Satellite-derived (Landsat)	[37, 38]
Normalized Difference Vegetation Index	National +	250 m	2000 to present	Satellite-derived (MODIS)	[39]
Normalized Difference Vegetation Index	National +	1 km	1979 to present	Satellite-derived (AVHRR)	[40, 41]
Green View Index	National	Postal code-specific	2017 only	Google Street View-derived	not published (or use MIT)
Climate and Weather					
Temperature metrics (daily, monthly, annual averages, ranges, event frequencies) and derived water balance metrics (potential and actual evapotranspiration)	National	10 km	1950–2010	Interpolated continuous surfaces based on observation station data	[42]
Temperature metrics (max, min, mean, heat deg. days, cool deg. days), total precipitation, snow on ground	National	N/A	1950- to present	National Climate Data and Information Archive - observation station data	[43]
Neighbourhood Factors					
Walkability	National	Postal code-specific	circa 2015	GIS-derived walkability (based on land use mix, street connectivity and population density)	[47]
Walkability	Regional	Postal code-specific	various specific years	GIS-derived walkability (land-use mix, residential density, and street connectivity)	[48]
Canadian Marginalization Deprivation Index	National	Census dissemination area	Census years 1991–2011	Derived from census variables	[49
Pampalon Deprivation Index	National	Census dissemination area	Census years 1991–2011	Derived from census variables	[50]
Nighttime light	National +	1 km	1992 to present	Satellite-derived from the US Defense Meteorological Satellite Program’s Operational Linescan System (DMSP-OLS) - annual average	[51]

Three air pollutants have been used most often in recent epidemiological research in Canada; fine particulate matter (PM_2.5_), nitrogen dioxide (NO_2_) and ozone (O_3_). National coverage for PM_2.5_ is derived from the recently developed 1 × 1 km satellite-derived PM_2.5_ surface [27]. Exposures for NO_2_ are estimated empirically from a national Land-Use Regression (LUR) model [28] and finer scale spatial patterns in NO_2_ are available from LUR models for 10 cities in Canada [29]. Exposures to O_3_ have been derived from a combination of observations and output from the chemical transport model developed by Environment and Climate Change Canada for air quality prediction and used in recent epidemiological studies [30, 31]. A national surface for sulphur dioxide (SO_2_) is also available based upon recent progress in satellite detection and extrapolation to surface concentrations [32]. Temporal coverage of CANUE national and urban-level air pollutant exposure surfaces will initially extend from 2000 to the present.

The LUR method has also been used to model spatial surfaces of urban environmental noise exposure in two Canadian cities, Montreal [33] and Toronto [34], with field monitoring also conducted in other cities (e.g., Vancouver, Ottawa, and Halifax [35]). Vancouver noise maps for 2003 have been generated using the deterministic propagation model CadnaA [36]. A similar model is currently being run for Montreal for 2008. CANUE is documenting these noise exposure surfaces to make them more-widely available for epidemiological research.

The Normalized Difference Vegetation Index (NDVI), which is derived from satellite measurements of near infrared and visible red radiation reflected by vegetation, is readily available as an indicator of greenness and the exposures this metric may represent. This includes already developed annual and peak growing season NDVI products using Landsat 5 and Landsat 8 [37, 38], the Moderate Resolution Imaging Spectroradiometer (MODIS) [39] or the Advanced Very High Resolution Radiometer (AVHRR) data which provides annual coverage and potentially seasonal variations as far back as 1981 [40, 41].

To facilitate research on how extreme weather and climate relate to the incidence of chronic disease, CANUE is including climate data. At present, the spatial resolution available across Canada is limited and originates from interpolation of the available, largely routine observations and/or from re-analysis products combining models and observations. As such, an observation-based dataset of daily maximum and minimum temperatures and precipitation produced by the Canadian Forest Service and Environment and Climate Change Canada is available at 10 × 10 km [42] and raw data can also be accessed by station [43] to derive proximity-based metrics of weather and climate (i.e., summary statistics based on nearest stations). The Climate Forecast System Reanalysis [44] or the Japanese 55-year Reanalysis [45] are comparable, while higher resolution observed gridded data, such as the ~800 m data covering British Columbia through the Parameter-elevation Regressions on Independent Slopes Model (PRISM) [46], are expected to become available nationally in the future.

Geographic Information Systems (GIS) provide the tools for calculation of a variety of exposure metrics at a fine scale across urban areas and within neighbourhoods. Walkability, for which multiple measures have been developed [47, 48], will be included early in the CANUE data holdings. The Canadian Census data includes socioeconomic data for the country from which several indices can be computed and mapped. The Canadian Marginalization Index (CanMarg) [49] and the Pampalon Index [50] have been or are being determined for multiple cycles of the Canadian census from the 1980s to the present. Light at night, which is derived from satellite observations with 1 km resolution, is also available and is listed as part of the neighbourhood factors domain [51].

### Building on the existing exposure information

Limitations associated with the exposure measures currently available for each domain are being addressed by the CANUE Working Groups. This involves the initiation of research projects and/or targeted workshops to guide future projects. Priorities for this work were developed at a national workshop held in December 2016 (www.canue.ca/workshop). Clearly, CANUE will not be able to address all limitations within five years. In Table 3, selected key exposure metric advances planned for this time period (i.e., through ~2021) are summarized and through new partnerships CANUE will be able to further expand the amount and type of new exposure data available for health research.

**Table 3:** Future Metrics

Future Metrics	Geographic Extent	Spatial Resolution	Time Periods	Description	Ref.
Air Pollution					
Surface concentrations of NO2, SO2 and CO	National +	7 km	2016 onward	New satellite and sensor, TropOMI, upgrading the current OMI measurements to provide increased spatial resolution with daily global coverage.	
Surface concentrations of PM2.5		2 km	2016 onward	GOES-R geostationary satellite observing aerosol optical depth on an hourly or better time resolution during daylight hours.	[72]
Surface concentrations of PM2.5, NO2, SO2	National +	5+ km	planned for 2019 onward	New geostationary satellite and sensor, TEMPO, upgrading the current OMI measurements and enhancing TropOMI with hourly or better time resolution during daylight hours; similar satellites planned for Europe (Sentinel-4) and Asia (GEMS).	[69–71]
Ambient concentrations of O3, PM2.5 and NO2	National (and city-specific)	10 km	Daily, monthly and annual starting in 2000	Operational forecast chemical transport model (GEM-MACH) with objective analysis for NO2, O3 and PM2.5 produced by Environment and Climate Change Canada; For NO2, additional chemical transport model (CTM) runs are being combined with local LUR models (‘hybrid approach’) by Health Canada. Where the LUR and CTM are combined the spatial resolution is ~50 m.	
Ambient concentrations of PM2.5 and NO2	National	<100 m	Annual	National empirical models using surface observations and multiple predictors from diverse sources such as satellites, CTMs, GIS, transportation models.	
Noise Pollution					
A-weighted sound pressure level and related summary metrics	National	<100 m	2017, with plans to adjust for historical estimates	New LUR model(s) to be developed based upon future noise measurements in selected Canadian cities.	
Greenness					
Metrics reflecting greenness accessibility and type (land use and land cover)	National	Neighbourhood-level	To be determined	Metrics to be identified by Greenness Working Group, and may include seasonal NDVI, measures of tree cover/canopy, tree species inventories at city scale, etc. and data from Sentinel-2 or Planet satellites.	
Climate and Weather					
Local Climate Zones	National +	Varies depending on landuse/cover	2017 with plans to adjust for historical estimates	Method to be developed and evaluated for using image classification and deep machine learning to map local climate zones based on building type, height, and vegetation.	[62–64]
Long term climate metrics	National +	32 km	1979 to present	Derived from Climate Forecast System Reananlysis data, metrics to be identified by Weather and Climate Working Group.	[44]
Long term climate metrics	National +	60 km	1958 to 2012	Derived from Japanese 55-year reanalysis, metrics to be identified by Weather and Climate Working Group.	[45]
Long term climate metrics	Regional (British Columbia)	800 m	Climate normal 30 year periods (1971–2000 and 1981–2010)	Derived from PRISM data, metrics to be identified by Weather and Climate Working Group.	[46]
Neighbourhood Factors					
Walkability	National		To be determined	New metrics to be developed reflecting age-specific and season-specific patterns and may consider landuse and landcover data; Representativeness of physical activity to be evaluated with surveys, GPS and accelerometry.	
Food environment	National			New metrics to be developed using a variety of information sources including GIS databases, ground-truth observations and Google StreetView.	
Transportation					
Car and truck volumes and traff emissions (CO, PM2.5, NOx, BC, selected VOCs)	Regional	Road segment	2016 - Halifax; 2006, 2011 - Montreal, Winnipeg; 1986, 2001, 2006 and 2011 - Toronto, Hamilton	Method development to be extended to other Canadian cities, and key input data for noise and air quality models.	

#### New exposure metrics and spatial surfaces

Transportation infrastructure is a key element of urban form (Fig. 2). There are multiple pathways through which it can affect health, from the resulting air and noise pollution to commute times and commuting mode choice to changes in active transportation behaviour. Therefore, improving Canadian urban scale data on transportation has potential benefits across domains. With this in mind, the Transportation Working Group is focusing on developing nationally consistent traffic volume and traffic emission maps. Initially this will include private vehicle travel behaviour for Canada’s three largest cities; Vancouver, Montreal and Toronto, as well as Halifax, Ottawa and Calgary. Maps have historically been limited for trucks i.e., goods movement; however, through CANUE, truck volumes and emissions will be generated for Halifax (a single year) and the Greater Toronto and Hamilton areas (4 separate years), enabling first ever maps for these cities of diesel emission patterns and potential exposures, relative to gasoline engine emissions, and applicable to urban populations. Depending on the level of success for this first set of cities and on the availability of input information for modelling private vehicle and truck flows, other cities will be added. Identification of areas of higher proportions of truck traffic versus automobiles will enable new research into the health effects of these major sources of near-road exposure potentially leading to more-informed transportation policies.

Another key function of CANUE is to facilitate interaction among Working Groups for consistency in development of exposure data, sharing of measurements and models, and to be better able to conduct integrated studies of urban form and health. For example, the Air and the Noise Pollution Working Groups are aligned with the Transportation Working Group to enable each to capitalize on the new traffic maps for development of improved exposure surfaces. Due to the limited amount of previous research, substantial gaps exist with regards to noise exposure in Canada (i.e., spatially-resolved exposure estimates are currently only available for disparate times for Montreal, Toronto and Vancouver). However, building upon experience from these three cities and improved traffic information from the Transportation Working Group, a consistent methodology for estimating noise exposure will be developed and applied for other major Canadian cities. Given that the application of noise dispersion models such as CadnaA to all of Canada or even all cities is not feasible, a land-use regression-based approach will be applied (Table 3). In parallel, a survey of existing field data will be conducted and an approach will be developed for adjusting the new national LUR model to represent historical noise levels.

National exposure surfaces and separate urban LUR models are relatively well-developed for air pollution. However, limitations remain and thus the Air Pollution Working Group aims to update the national exposure maps for PM_2.5_, NO_2_ and O_3_. The currently available maps were generated independently, with differences in methodology and temporal coverage. For example, the NO_2_ surface includes the influence of near-road exposures [31] while PM_2.5_ and O_3_ do not. To address inconsistencies and/or to improve the current exposure estimates, two different approaches are being followed. The first is based upon chemical transport models. Hourly output from the current Environment and Climate Change Canada (ECCC) operational chemical transport model - the Global Environmental Multi-scale – Modelling Air Quality and Chemistry (GEM-MACH) - which is combined with surface observations using an objective analysis approach [52], is being provided to CANUE for development of exposure metrics. This approach is being further developed by Health Canada to provide finer scale exposure estimates for NO_2_ by combining the chemical transport model with LUR models in a ‘hybrid approach’. The second approach is to update the national NO_2_ and PM_2.5_ surfaces, which were empirically derived, through inclusion of larger amounts of data, including near-road conditions, and use of new methods (e.g., machine-learning) in the model development.

Improvements in NDVI spatial resolution and development of more health-relevant greenness exposure metrics are being pursued through CANUE to advance their utility. The integration of land use and land cover data, biophysical measures of greenness such as tree canopy cover, tree species data and NDVI seasonality is being undertaken in order to explore how this approach could lead to more accurate or representative greenness metrics. Furthermore, increases in NDVI resolution to better than 30 m may be feasible using a combination of Planet images [53] and Landsat 8 data. The potential of Sentinel-2 [54] multispectral imagery for providing frequent (up to every 5 days) land use and land cover mapping, greenness and leaf area index at high spatial resolution is also being explored.

NDVI by itself does not directly capture salient aspects of the links between greenness and health outcomes. Thus, other related metrics have employed additional landcover information (i.e., percent canopy cover) and/or land use information (park boundaries, accessibility via transportation networks) [55, 56] in attempts to address this limitation. The CANUE Greenness Working Group is conducting a review to inform future development of a larger suite of metrics that will reflect the underlying features associated with greenness that could impact health. For example, proximity to greenness could influence physical activity levels within the population if the areas observed to be ‘green’ contain certain infrastructure like walking paths.

Independent, but related to greenness is walkability. Associations between walkability and health outcomes such as obesity, cardiovascular health, and physical activity have been observed in many regions of the developed world [57, 58]. Similar observations have been made relating neighbourhood food environments, although not consistently across regions and among countries [59]. Over the past several decades, many methods have been used for quantitatively characterizing aspects of walkability and food environments [60, 61]. The Neighbourhood Factors Working Group within CANUE is leading a review of extant metrics with a focus on identifying those that are applicable in Canada and can be implemented nationally, given large geographic and seasonal differences, and varying behaviours by age.

Urban morphology interacts with climate and extreme weather creating local conditions that can potentially impact population health. The sensitivity of the currently available meteorological or climatological data to these interactions is limited due to their complexity and the spatial resolution of the data. The local climate zone (LCZ) framework, which uses urban morphology characteristics to estimate the magnitude of the urban heat island and other hazards [62], will be assessed by the Climate Working Group for its utility in health research. LCZs were originally developed to characterize the environment surrounding meteorological field sites to better account for urban influences on observed temperature [63]. Factors evaluated include built types (i.e., compact high-rise buildings, sparsely built, industrial, etc.) and land cover types (i.e., dense trees, low plant, water, etc.). Currently, the World Urban Database and Access Portal Tools (WUDAPT) project is facilitating mapping LCZs using Google Earth and crowd-sourcing techniques. City-specific volunteers around the world [64] are providing valuable local-scale observations to reliably map LCZs. Through CANUE, LCZs will be developed for all of Canada, and then linked to air quality, vegetation, aeroallergen exposure, urban flooding, and other hazard indicators as well as future climate conditions, to assess how the LCZ framework can inform environmental health studies.

#### Increases in the volume, variety and velocity of big environmental data

A range of new data sources have the potential to greatly increase the quantity of the environmental exposure data available for health research. Satellite-based measurements of spatial patterns in a variety of physical and chemical features at the Earth’s surface have been of tremendous value to a wide range of disciplines. However, the amount of data collected with each satellite overpass or image is a challenging big data stream to manage. In the study of atmospheric trace gases and aerosols, satellite measurements, which have come of age in the last two decades, have been highly beneficial. Estimates of chronic exposure to air pollution are now possible for much of the globe [65] leading to improved characterization of exposure-response relationships [66, 67] and estimates of the role of particulate air pollution in the global burden of disease [68] .

**Fig. 3:**
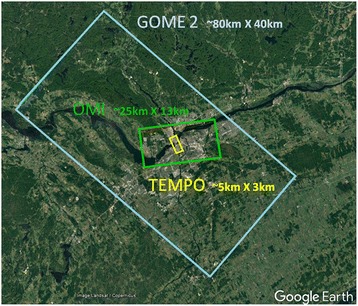
Relative differences in spatial resolution of trace gas measurements (e.g., NO_2_) from satellite-based measurements over Ottawa, Canada. Rectangles show the minimum sizes areas covered (pixel size) with three generations of satellites. The blue square corresponds to the less than daily observation frequency of GOME 2. The green square, the daily frequency OMI measurements and, the daylight, hourly frequency of TEMPO (yellow square). The new TEMPO satellite will be capable of collecting data in the ultraviolet and visible wavelengths at approximately 2 km × 5 km spatial resolution. Once in operation TEMPO will produce data for approximately 2.5 million grid cells every daylight hour, equivalent to 1 terabyte of data daily

The volume and velocity and the potential variability and value of satellite air pollution measurements are expected to increase substantially during the first five years of CANUE’s program with the launch of new geostationary satellites. The Tropospheric Emissions: Monitoring of Pollution instrument (TEMPO) [69], Geostationary Environment Monitoring Spectrometer (GEMS) [70] and Sentinel-4 [71], for North America, Asia and Europe, respectively, will provide daytime hourly observations with increased spatial resolution compared to the previous satellites providing information on trace gases in the troposphere (Fig. 3). The full potential of this new big data stream cannot be fully appreciated, but for chronic and even sub-acute exposure estimation going forward into the 2020’s these satellites and, the new Geostationary Operational Environmental Satellite-R series (GOES-R) [72] satellites enhancing information on aerosol optical depth (PM_2.5_), will represent the state-of-the-art. CANUE is developing the infrastructure and algorithms to be able to capitalize on these data for environmental health research and monitoring. Given the new data streams becoming available it may be feasible in the future to link the temporal and spatial patterns in urban NO_2_ and PM_2.5_ levels from the geostationary satellites to traffic flow patterns derived from tracking mobile phone locations (from global positioning systems or tower signals) leading to new understanding of the dynamic between urban form, traffic, air pollution, and ultimately health. Mobile phone data are already being used to refine air pollution exposure estimates by tracking population movements during the day [73, 74].

Climate, weather and air quality forecasting models are another source of big data with potential value in environmental health research. The GEM-MACH model and its objective analysis product, described above (Table 3), has provided data for national ground-level O_3_ exposure estimates (Table 2) [30]. CANUE is collaborating with ECCC to make data from 2013 to the present available for a variety of exposure time windows. This modelling system currently produces a large volume of data year-round at 10 km resolution across North America. Methods are being developed through CANUE to routinely capture data on hourly ozone, PM_2.5_ and NO_2_ concentrations in near real-time and preparing exposure-relevant variables. Future versions of the model and objective analysis product will likely increase spatial resolution (e.g., 2.5 km) leading to larger volumes of data and potentially better exposure precision. Ultimately, air quality researchers expect to integrate the hourly satellite data with these modelling tools to further improve accuracy. Such advances have the potential to benefit environmental health research far into the future.

The meteorological models that support weather forecasting and are essential to the air quality modelling represent another big environmental data stream of potential value to health research. In the near future these models are expected to be capable of resolving urban scale features leading to more-realistic characterization of climate phenomena such as heat islands. Such output, which CANUE is aiming to utilize in partnership with OURANOS [75], will support future research exploring how current and future climate and extreme weather events impacts public health. New knowledge in this area could help Canada’s urban areas prepare for climate change (i.e., adaptation to build resiliency).

Google Earth Engine [76] was introduced in 2010 to enable the global-scale monitoring and measurement of changes in the environment. The ‘Earth Engine’ provides two key functions: 1) the curating and management of historical and ongoing satellite data; and 2) an easy to use analytical platform that allows researchers to create and implement scripts and algorithms to process the data into useful metrics of environmental characteristics and their change over time. For example, with annual 30 m NDVI data from Landsat in Google Earth Engine for 1984 onward it will be possible to generate greenness exposure maps or maps of areas of urban development (e.g., road coverage) at a spatial resolution, temporal coverage and geographic extent not easily accomplished without the big data functionality of Google Earth Engine. This temporal information has the potential to improve exposure estimates for cohorts by integrating over a larger portion of each individual’s lifetime especially if residential history data can be obtained.

Useful metrics of green canopy coverage, which is relevant to urban heat and likely a range of other issues (e.g., aeroallergens), have recently been shown to be computationally feasible from Google Street View images [77], and are comparable to audits conducted by direct observation [78]. While this virtual audit saves time and money and it is repeatable among different observers, automation could lead to even greater savings and consistency, also generating large amounts of data from which to derive exposure metrics. There is a rapidly growing literature illustrating the automation of index calculations using Google Street View, for example, a Green Vegetation Index (GVI) [79]. CANUE will explore a street level-based greenness indicator in the near term and continue to refine and develop new methods and indicators using available imagery.

Prospectively, new tools being developed for collecting mobility data to inform transportation planners, including smart-phone applications, which collect GPS coordinates to infer locations, movement, mode of transportation and activity can be used to determine individual activity-travel diaries [80]. These ‘apps’ could be adopted for use in large cohorts (e.g., Canadian Partnership for Tomorrow Project [15]) to obtain mobility data for tens of thousands of subjects. They could also be enhanced to prompt, in a minimally burdensome fashion, for longitudinal information related to personal behaviours (e.g., recent meals and exercise) and to process measurement data from sensors in the phone (e.g., accelerometer, microphone) or from companion sensors to improve exposure assessment. Considerable effort is currently being focused in this area (e.g., The Pediatric Research using Integrated Sensor Monitoring Systems (PRISMS) [81]; and, while not the primary focus of CANUE, measurement sub-studies exploring youth physical activity are being planned to support some of Canada’s active birth cohorts (e.g., Canadian Healthy Infant Longitudinal Development Study (CHILD) [82]).

### Challenges

Key challenges for large environmental health studies, particularly those aiming to implement an exposome-based approach, continue to be enrichment of cohorts with individual-level exposures, harmonization across cohorts and, and ultimately identification of modifiable risk factors leading to interventions that have benefits on population health. To help meet these challenges Stingone et al. [83] suggested that exposome studies would be well-served by centralized support and coordination to ensure that potential exposure assessment strategies are rigorously evaluated. CANUE represents an attempt to meet these challenges with respect to exogenous factors and, while CANUE is the largest coordinated effort in Canada around environmental exposure data, many challenges remain.

There is a long-standing need to better understand temporal change in spatial exposure patterns going back decades and how this contributes to exposure misclassification and subsequent epidemiological results [84, 85]. Detailed characterization of spatial patterns with high resolution that are indicative of chronic exposure is typically only accomplished for ‘snapshots’ in time because of the effort and expense required. It is therefore necessary to estimate temporal changes in these spatial exposure patterns by extrapolation of the spatial detail. This could include estimates covering longer time periods (i.e., decades) or particular months to years before or after the time of the ‘snapshot’. For air pollution a variety of extrapolation approaches have been used [84–87]; however, in order to have reasonable confidence in the estimates it is necessary to have monitoring site data with temporal coverage for the time periods and pollutants of interest and ideally from multiple locations depending upon the size of the spatial domain modelled. This is problematic because long-term exposures over relatively large geographic areas require estimates much further back in time pre-dating the monitoring of some pollutants (e.g., PM_2.5_). In these cases, there is likely much greater uncertainty in the exposure estimates [85], but they are difficult to quantify given lack of evaluation data.

The need for temporal extrapolation and uncertainty arising from lack of historical exposures are limitations impacting most of the exposure domains of interest to CANUE. The noise pollution maps are available for a limited number of cities and specific snapshots in time. New noise maps to be developed through CANUE will also face this limitation and their applicability to other time periods or longer time windows relies on the assumption of temporal stability. Given that a key source of noise is traffic and other transportation activities (e.g., airports) and the infrastructure for these is stable over relatively long periods, extrapolation is reasonable. However, road, air and train movements have changed overtime as well as emissions; the locations of many other noise sources can change more-rapidly; and even changes such as construction of noise barriers will alter exposure patterns. Further, fitting noise models to similar geospatial predictors as air pollution contributes to collinearity hindering attempts to isolate effects due to these two exposures [88].

Coordination through CANUE offers promise that some progress on these and other challenges can be achieved. Google’s Earth Engine, for example, is hypothesized to facilitate the analysis of big geospatial data with a temporal coverage that will be informative of changes in urban environment exposure metrics going back into the 1980s. CANUE provides the critical mass to explore this idea. Given high resolution surfaces of noise and air pollution, other health-relevant neighbourhood features and maps of local climate zones that indicate potential for heat islands, it may be possible, using local land use variables as model inputs, to develop algorithms that can relate land use classifications derived from the 30 m Landsat images. These algorithms, if robust and mechanistically-based, could then enable reliable estimation of a variety of urban form exposure variables back to 1984.

Residential mobility is also an important cause of misclassification when exposure assessment relies on geographic location. Often exposure is based upon a single home address, such as might be acquired at time of study recruitment or baseline. The potential for differential exposure misclassification has been demonstrated in birth cohorts [89], and can be expected to increase the longer the follow-up period or the longer the exposure time window of interest. Crouse et al. [30] reported that nearly 50% of the Canadian population moved at least once in the 5 year period from 2001 to 2006. They accounted for residential mobility during the 16 year of follow-up of Canadian Census Health and Environment Cohort and found that this led to larger hazard ratios compared to those determined using exposures assigned using a single baseline address. This attenuation in hazard ratio was greatest for NO_2_, less for PM_2.5_ and negligible for O_3_.

Residential history on study individuals, if available, can be used to determine time-weighted exposures, assuming exposure data are available for the different addresses reported. Ideally, such information is obtained in prospective cohorts through questionnaires. In practice this is not always the case and/or the data are incomplete. Administrative data housed at the federal and provincial level represents a different option, taking the burden away from the subjects, while standardizing the approach. CANUE is working with Statistics Canada through the Social Data Linkage Environment [90] to obtain annual residential history data for individuals in some cohorts following the method used by Crouse et al. [30]. Provincial health care records also retain addresses and these data are being assessed for reconstruction of residential history.

Daily mobility and time spent indoors poses another challenge for exposure assessment. Accounting for time at work or school and proximate exposures is feasible given sufficient information and resources. While where a person lives plays a major role in their relationship with all of the urban form features related to CANUE’s exposure domains, all locations where significant time is spent, including in transit (i.e., commuting), are potentially important (Fig. 2). CANUE is aiming to provide exposure metrics for many potential locations allowing for additional time-weighting of outdoor exposures. However, reliable time activity behaviour at the individual level represents a key limitation.

## Discussion

CANUE is compiling a wide range of geospatial datasets of exposure metrics that are known to be or hypothesized to be relevant to public health. However, these postal code specific metrics are just that; metrics that act as surrogates for more complex underlying processes that manifest as a health effect, adverse or beneficial. It is critical that we understand these processes as much as possible and consider whether the metric or surrogate being used is appropriate and ultimately informative of the root causes. Consequently, one criterion for CANUE’s efforts in refining exposure metrics is to improve their ability to reflect the underlying processes or mechanisms and to better understand these relationships. Through this approach we aim to improve our understanding of the uncertainties in the exposure metrics, which continue to be difficult to quantify. Furthermore, future studies involving multiple, interacting exposures can then be more informative.

The body of research is relatively large for impacts of single air pollutants or urban form characteristics such as greenness or walkability in isolation. There is less research evaluating different features of the urban form or exposures in combination [91–93]. Clearly, there is the potential for joint as well as counter-acting effects. For example, current understanding suggests that in countries with moderate to low air pollutant levels (e.g., Canada) the benefits of active transportation (i.e., physical activity) far outweigh the dis-benefits of enhanced air pollution exposure from greater inhalation rates [94]. Furthermore, transportation polices that reduce air pollution and increase active transportation are estimated to have large economic benefits [95]. However, these examples are based upon risk analysis using current epidemiological data, while the original epidemiological studies have tended to explore exposures separately. With CANUE facilitating linkage of air pollutant exposures and metrics related to physical activity, as well as other exposures (e.g., noise, stress associated with neighbourhood factors), to individuals cohorts, future epidemiological studies may be able to assess the effect of interactions in different regions of Canada with different socioeconomic and climatic conditions and for different members of the population.

CANUE will also focus on data that are available internationally, such as those derived by satellite instruments or global data collection initiatives such as those conducted by Google. By building on existing methods for deriving useful exposure metrics, implementing them nationally, and sharing newly developed methods using widely available input data, CANUE has the potential to contribute significantly to advancing environmental health studies globally. Making a wide variety of standardized metrics available will increase the comparability among studies, and potentially support the formation of very large virtual cohorts by combining results of studies from multiple countries. The statistical power these meta-studies may be capable of achieving is likely key to understanding the subtle interactions among environmental exposures related to urban form [87].

CANUE’s potential impact is based in large part on the willingness of its members to share methods and in some cases, proprietary input data or already-developed exposure metrics suitable for a national platform. CANUE is positioned as a neutral data broker, providing standardized metadata for each shared dataset, as well as a formal data sharing agreement with terms set by the data developer. Exposure data will be provided to established cohorts and administrative data holders, who then follow their own standard approval processes for providing access to both the confidential health data and related, and where possible, pre-linked exposure data. The challenges of working with multiple data developers, data sharing requirements, and varying capacities and procedures for data integration by health data holders are complex, but not insurmountable.

CANUE’s protocol for establishing a centralized, coordinated effort in deriving and linking urban-related environmental exposures to Canada’s wealth of cohorts and administrative health data holdings will increase efficiency by reducing duplication and insuring consistency in the exposure measures used. As such, CANUE will enable a more focused effort on filling gaps in exposure information, improving the range of exposures quantified, their precision and mechanistic relevance to health. Epidemiological studies will thus be better able to harness big environmental data in order to explore the common theme of urban form and health in an integrated manner, ultimately contributing new knowledge informing policies that enhance healthy urban living.

## Abbreviations

AVHRR: Advanced very high resolution radiometer
BC: Black carbon
CanMarg: Canadian marginalization index
CANUE: Canadian Urban Environmental Health Research Consortium
CHILD: Canadian Healthy Infant Longitudinal Development Study
CIHR: Canadian Institutes for Health Research
CO: Carbon monoxide
ECCC: Environment and Climate Change Canada
GEM-MACH: Global Environmental Multi-scale – Modelling Air Quality and Chemistry
GEMS: Geostationary Environment Monitoring Spectrometer
GIS: Geographic information systems
GOES-R: Geostationary Operational Environmental Satellite-R series
GVI: Green vegetation index
LCZ: Local climate zone
LUR: Land use regression
MODIS: Moderate resolution imaging spectroradiometer
NDVI: Normalized Difference Vegetation Index
NO2: Nitrogen dioxide
NOx: Nitrogen oxides
O3: Ozone
PM2.5: Fine particulate matter
PRISM: Parameter-elevation regressions on independent slopes model
PRISMS: Pediatric research using integrated sensor monitoring systems
SO2: Sulphur dioxide
TEMPO: Tropospheric Emissions: Monitoring of Pollution instrument
VOCs: Volatile organic compounds
WUDAPT: World Urban Database and Access Portal
